# Environmental Effects of Moisture and Elevated Temperatures on the Mode I and Mode II Interlaminar Fracture Toughness of a Toughened Epoxy Carbon Fibre Reinforced Polymer

**DOI:** 10.3390/polym17111503

**Published:** 2025-05-28

**Authors:** Anna Williams, Ian Hamerton, Giuliano Allegri

**Affiliations:** Bristol Composites Institute, University of Bristol, Queen’s Building, University Walk, Bristol BS8 1TR, UK; ian.hamerton@bristol.ac.uk (I.H.); giuliano.allegri@bristol.ac.uk (G.A.)

**Keywords:** interlaminar fracture toughness, environmental effects, hot wet, fractography, carbon/epoxy

## Abstract

The use of composite materials within extreme environments is an exciting frontier in which a wealth of cutting-edge developments have taken place recently. Although there is vast knowledge of composites’ behaviour in standard room temperature and humidity, there is a great need to understand their performance in ‘hot/wet’ conditions, as these are the conditions of their envisaged applications. One of the key failure mechanisms within composites is interlaminar fracture, commonly referred to as delamination. The environmental effects of moisture and elevated temperatures on interlaminar fracture toughness are therefore essential design considerations for laminated aerospace-grade composite materials. IM7/8552, a toughened epoxy/carbon fibre reinforced polymer, was experimentally characterised in both ‘Dry’ and ‘Wet’ conditions at 23 °C and 90 °C. A moisture uptake study was conducted during the ‘Wet’ conditioning of the material in a 70 °C/85% relative humidity environment. Dynamic mechanical thermal analysis was carried out to determine the effect of moisture on the glass transition temperature of the material. Mode I initiation and propagation fracture properties were determined using double cantilevered beam specimens and Mode II initiation fracture properties were deduced using end-notched flexure specimens. The effects of precracking and the methodology of high-temperature testing are discussed in this report. Mode I interlaminar fracture toughness, $G_{IC}$, was found to increase with elevated temperatures and moisture content, with $G_{IC}=0.205\;{\mathrm{kJ}/m}^{2}$ in ‘Dry 23 °C’ conditions increasing by 26% to $G_{IC}=0.259\;{\mathrm{kJ}/m}^{2}$ in ‘Wet 90 °C’ conditions, demonstrating that the material exhibited its toughest behaviour in ‘hot/wet’ conditions. Increased ductility due to matrix softening and fibre bridging caused by temperature and moisture were key contributors to the elevated $G_{IC}$ values. Mode II interlaminar fracture toughness, $G_{IIC}$, was observed to decrease most significantly when moisture or elevated temperature was applied individually, with the combination of ‘hot/wet’ conditions resulting in an 8% drop in $G_{IIC}$, with $G_{IIC}=0.586\;{\mathrm{kJ}/m}^{2}$ in ‘Dry 23 °C’ conditions and $G_{IIC}=0.541\;{\mathrm{kJ}/m}^{2}$ in ‘Wet 90 °C’ conditions. The coupled effect of fibre-matrix interface degradation and increased plasticity due to moisture resulted in a relatively small knockdown on $G_{IIC}$ compared to $G_{IC}$ in ‘hot/wet’ conditions. Fractographic studies of the tested specimens were conducted using scanning electron microscopy. Noteworthy surface topography features were observed on specimens of different fracture modes, moisture saturation levels, and test temperature conditions, including scarps, cusps, broken fibres and river markings. The qualitative features identified during microscopy are critically examined to extrapolate the differences in quantitative results in the various environmental conditions.

## 1. Introduction

The replacement of metals with advanced composite materials has become commonplace within a range of industries [1]. Thus, elevated strength, high modulus-to-weight ratio, and an array of manufacturing processes have led to growing composite use in the aerospace, automotive, and civil industries. The composite industry has seen vast improvements in the last few decades, with the development of reliable manufacturing methods, standardised experimental testing and rigorous characterization [2]. High-temperature composites have been introduced in recent years to allow for the use of composite materials in conditions where harsher environmental effects are of concern. This has led to the formation of high-temperature organic matrices, which can operate in temperatures greater than 100 °C and possess processability, formability, and ductility not found in inorganic matrix materials such as ceramics. Therefore, thermoplastic and thermoset resins have been developed to maintain their properties at higher temperatures [3,4,5,6]. Although composites containing epoxy resins have dominated the aircraft market since the 1970s, and alongside aluminium are the most common structural air-frame material [7], their applications are usually restricted to structures which do not experience elevated temperatures. There is a push to use organic matrix composites (OMCs) within a wider range of aircraft components, which, in turn, requires knowledge of their potential to fail at elevated temperatures and in moisture-rich environments.

The exposure to harsh environments, including higher temperatures and moisture-saturated conditions, leads to the accelerated degradation of composite materials. This environment has coined the name ‘hot/wet’, and materials classified to be in this ‘hot/wet’ environment are assumed to be in their most severely degraded condition [8]. Aceti summarises the historically documented effects of hygrothermal ageing within composite parts, in which exposure to moisture and thermal cycling has been a leading cause of component failure [9]. The deterioration of material properties, including a decrease in tensile, compression, and shear strength, caused by the rapid uptake of water molecules into composite materials has been reported [10,11]. One of the key properties of interest when designing composite materials for ‘hot/wet’ environments is interlaminar fracture toughness [12]. Most OMC manufacturing processes involve stacking layers of fibres pre-impregnated with resin, which may result in stresses or voids developing between layers (i.e., laminae). Mode I interlaminar fracture, commonly referred to as the opening mode or delamination, involves interlaminar separation and is the primary failure mechanism within composite structures [13]. Mode II interlaminar fracture, known as the sliding mode, involves a failure in plane-shear between layers. A material’s resistance to opening and shear delamination is calculated by determining its strain energy release rate for delamination growth [14,15].

Despite the predominantly observed deterioration of properties caused by ‘hot/wet’ environments, owing to the fundamental mechanisms involved in delamination, both temperature and moisture have been seen to increase Mode I interlaminar fracture toughness [16,17]. There have, however, been an array of disparities in reported Mode I interlaminar properties when testing at elevated temperatures and in different relative humidities independently. Studies dating back to the 1980s have recorded an increase [18,19,20,21,22], a decrease [23,24,25] and no significant change [26,27] in Mode I interlaminar fracture toughness when temperature or moisture was applied. In contrast, Mode II interlaminar fracture toughness tends to decrease in ‘hot/wet’ conditions [16,17]. Studies that involve only the application of heat [21,23] and only the administration of moisture [18,19,23,25,28] also show the reduction in Mode II interlaminar fracture toughness.

The noted disparities in recorded ‘hot/wet’ interlaminar properties and sparse existing ‘hot/wet’ data highlight the importance of characterising OMCs in more extreme environments when exposed to temperature and moisture. There is a vast range of ever-growing potential resin and fibre combinations, each of which has unique interlaminar properties. As industries push towards integrating composites into more structures, to reduce weight in the constant drive towards a more sustainable future, the fracture properties of each new material combination must be thoroughly understood. This work contributes to limited data found in the literature by characterising a commonly-used commercial carbon/epoxy composite, IM7/8552, in ‘Dry’ and ‘Wet’ conditions, at room temperature (nominally 23 °C) and 90 °C. The effect of conditioning in heat and moisture on the materials’ moisture uptake and glass transition temperature is explored. The ‘hot/wet’ interlaminar properties are calculated and compared for different test conditions and fracture modes. An in-depth analysis of the specimen surfaces post-fracture is carried out using scanning electron microscopy to draw qualitative comparisons and highlight the effects of heat and moisture on the material. A detailed procedure ranging from specimen manufacture to post-test imaging is outlined to aid the understanding of how the results in this work were conceived.

## 2. Materials, Manufacturing, and Conditioning

### 2.1. Material Selection

Unidirectional carbon epoxy prepreg IM7/8552 (Hexcel Composites, Duxford, UK) was used in this study. Table 1 shows the mechanical properties reported by Hexcel [29]. IM7/8552 is a well-established aerospace-grade, toughened composite material. The matrix, HexPly^®^ 8552 Epoxy Resin (Hexcel Composites, Duxford, UK), is a thermoplastic toughened high-performance amine-cured epoxy resin system which has high impact resistance and damage tolerance [30]. IM7/8552 composite parts are manufactured by laying up cut plies and curing under heat and pressure using an autoclave. It can operate in temperatures up to 121 °C and is available on multiple reinforcements. IM7 fibres are continuous, intermediate modulus PAN-based fibres which are surface treated and sized for compatibility with the toughened epoxy matrix [31]. A comparable system is T300/977-2 (Syensqo, Brussels, Belgium). It also contains a toughened epoxy matrix and carbon fibres, but with slightly lower $T_{g}$ and strength properties [32,33]. Ultimately, IM7/8552 was chosen for testing as it has been established as a favoured product for aerospace structures.

### 2.2. Specimen Manufacturing

Dual cantilever beam (DCB) specimens were used for Mode I interlaminar fracture testing [14], and end-notched flexure (ENF) specimens were used for Mode II interlaminar fracture testing [15]. IM7-8552 has a nominal cured ply thickness of 0.131 mm. Plies at 0° orientation were used throughout except for ±5° for the central plies of non-precracked tests. The laminates were manufactured, with a debulking process applied after every 4 plies to consolidate the layers. At the mid-plane of each laminate, a 12 μm non-adhesive polytetrafluoroethylene (PTFE) insert was implanted to form the crack initiation location, on the plane of delamination. Once stacked, the laminates were placed on an aluminium tooling plate and appropriately bagged for autoclave curing. The prescribed curing cycle for IM7/8552 was followed to produce the cured laminates. Specimens were cut from the laminates to produce 140 mm by 20 mm by 3 mm DCB specimens and 170 mm by 20 mm by 3.5 mm ENF specimens, using the dimensions outlined in the ASTM standards [14,15]. The correct orientation of the laminate during cutting was essential to ensure that the pre-inserted crack was in the right location and the fibres ran along the length of the specimens.

### 2.3. Conditioning and Preparation

All specimens were placed into a 40 °C drying oven for 10 days until they reached a weight change of less than 0.01% between two consecutive days. The specimens were then classified as ‘Dry’. Half of the specimens were sealed in a bag for ‘Dry’ testing and the other half were placed into a humidity-controlled environmental chamber. The chamber contained a humidity of 85% and a temperature of 70 °C, following ASTM D5229 standard for moisture absorption and conditioning equilibrium for polymer matrix composite materials [34]. A specimen, known as a ‘traveller’ specimen, was taken out on various days and weighed to measure moisture uptake. The specimens remained in this chamber until a weight change of less than 0.01% between two consecutive days was recorded. These specimens were saturated at a moisture fraction by weight of ≈1%, which classified them as ‘Wet’, removed from the conditioning chamber, and placed in a sealed bag until specimen preparation commenced.

**Figure 1:**
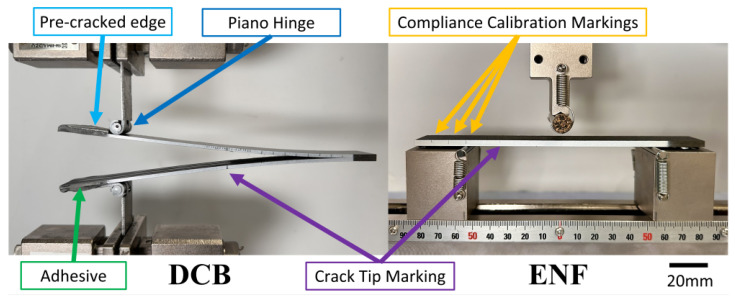
A DCB Specimen (ASTM D5528) [14] and an ENF Specimen (ASTM D7905) [15] loaded in their respective test rigs.

A layer of white spray paint was applied along the edge of the specimens. On the DCB specimens, the edge of a calliper was used to mark the location 75 mm from the precracked edge to note the corresponding initial delamination length of 50 mm. Further markings were made at regular intervals for crack tracking. A custom 3D-printed guide was used to align the specimens with the piano hinges. A thin layer of an epoxy adhesive, Araldite 2014-2 (RS Components), was used to bond the specimens and the piano hinges together. A post-cure at 80 °C was carried out to increase the bonding strength of the Araldite, crucial for high-temperature testing. The ‘Dry’ specimens were post-cured in an oven, and the ‘Wet’ specimens were post-cured in the environmental chamber to minimise moisture loss. On the ENF specimens, compliance calibration lines were marked at intervals of 20 mm, 30 mm, and 40 mm from the crack tip. Figure 1 shows DCB and ENF specimens which have undergone all the required preparation for testing.

## 3. Experimental Set Up and Applied Test Methods

**Figure 2:**
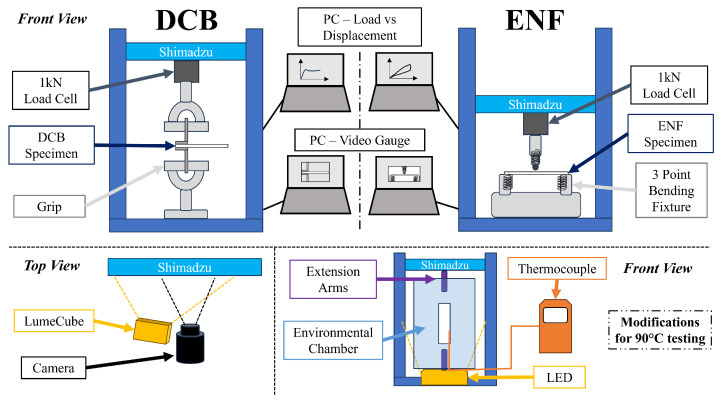
Room and elevated temperature test set up schematic used for Mode I (DCB) and Mode II (ENF) interlaminar fracture testing.

An Autograph AGS-X Series Universal 10N-10kN Test Frame (Shimadzu, Tokyo, Japan) fitted with a 1 kN load cell was employed to carry out Mode I and Mode II fracture toughness testing. A schematic of the test set-up using DCB and ENF specimens is shown in Figure 2.

### 3.1. Precracking

Half of all the DCB specimens were precracked before testing. Loading and unloading were carried out at a fixed rate of 2 mm/min to minimise specimen damage. Specimens were classified as precracked when a crack propagation length of between 2 mm and 5 mm had been reached. To obtain the precracks, DCB specimens were loaded in tension and the locations of the new crack tips were marked after the specimens were unloaded. ENF specimens were precracked via a custom-made 3D-printed slim wedge. For the ENF specimens, new compliance calibration markings were made according to the location of the new crack tip.

### 3.2. Test Procedure

DCB specimens were fixed into grips via the piano hinges and loaded in tension at a displacement-controlled rate of 2 mm/min, as specified by ASTM D5528 [14]. The load force and vertical displacement of the test machine were recorded throughout the test until a manual stop was implemented at a crack propagation length of 50 mm. An LED light (LumeCube, Carlsbad, CA, USA) was used to illuminate the crack front. An iMetrum video gauge (Bristol, UK) was used throughout the entire test, capturing at a rate of 1 frame per second, to record the vertical displacement of the specimen and the crack propagation as the force was applied. ImageJ (FIJI) was used to search through the video files, frame by frame, to record the propagation length of the crack tip. Force, vertical displacement, time, and horizontal delamination length data collected throughout the test were entered into an in-house MATLAB R2022b script for data analysis. The specimens were unloaded at the same rate as loading to ensure the fracture surface was not modified.

ENF specimens were placed in a three-point bend fixture, and a compressive force at a rate of 0.5 mm/min was applied to the centre of the specimens. The compressive load force and vertical displacement of the test machine were recorded until the specimen fractured, resulting in a load drop which triggered the end of the test. Crack tracking video equipment was not used in Mode II testing as crack propagation was unstable and unmeasurable at the given frame rate. The specimens were unloaded at the same rate as loading to preserve the interlaminar fracture surface.

### 3.3. Hot/Wet Testing

Modifications to the test set-up were made to allow high-temperature testing to take place. A TCE-N300-CE Thermostatic Chamber (Shimadzu, Tokyo, Japan) was used to raise the test temperature to 90 °C. A thermocouple was used to verify the specimen temperature. Elevated temperature testing commenced when the temperature of the specimen had reached equilibrium. The light source was changed to a high-powered LED, angled to reflect into the bottom of the chamber. Owing to the small viewing window on the door of the thermostatic chamber, the video gauge was angled to allow full crack propagation to be captured.

The ‘Dry’ and ‘Wet’ specimens were tested at room temperature (i.e., 23 °C) and at high temperature, 90 °C. The ‘Dry’ specimens were taken out of the sealed bag and tested. Fully prepared ‘Wet’ DCB specimens remained in the environmental chamber until they were directly transferred into the test fixture. ‘Wet’ specimens were weighed before and after high temperature testing, and a weight loss of less than 5% was recorded. The four test conditions are ‘Dry 23 °C’, ‘Dry 90 °C’, ‘Wet 23 °C’, and ‘Wet 90 °C’. There were at least three repetitions at each test condition.

### 3.4. Data Reduction

Mode I fracture toughness, $G_{IC}$, is determined using a DCB specimen, according to ASTM D5528 [14]. It measures the strain energy release rate,

(1) $$G=\frac{-1}{b}\frac{dU}{da},$$

where *b* is the sample width, *a* is the delamination length and *U* is the elastic strain energy in the specimen. Simple Beam Theory is employed to calculate the Mode I fracture toughness of the material,

(2) $$G_{I}=\frac{3P\delta_{c}}{2ba},$$

where $P_{c}$ is the applied critical load and $\delta_{c}$ is the critical vertical displacement of the beam. The Modified Beam Theory is the most conservative method for calculating $G_{IC}$ and is used to account for the rotation at the boundary condition, which is assumed to be fixed in Simple Beam Theory. To achieve this, a correction factor, Δ, is calculated to take into account the compliance of the beam. $P_{c}$ was determined from the intersection point of the load displacement curve and the compliance line offset by 5%. This gives the modified equation for Mode I fracture toughness,

(3) $$G_{IC}=\frac{3\;P_{c}\;\delta_{c}}{2b(a+|\Delta |)},$$

which is the selected data reduction method within this work to determine the interlaminar fracture toughness of the DCB specimens. Mode I Propagation Interlaminar Fracture Toughness, $G_{IP}$, was calculated using a modified version of Equation (3), where *P* and δ were obtained from the load displacement plots as the change in crack length, noted by Δa, was visibly identified.

Mode II interlaminar fracture toughness, $G_{IIC}$, is determined using end-notched flexure (ENF) specimens under shear loading, following ASTM D7905 [15]. It utilises the same loss of strain energy shown in Equation (1). The compliance calibration points are marked for three delamination lengths, and the compliance, *C*, is calculated at each point using a linear least squares regression analysis to find the slope of the collected displacement and force data, according to the standard [15]. Compliance is then fitted against delamination length cubed,

(4) $$C=A+ma^{3},$$

where *A* is the intercept and *m* is the slope obtained from the compliance fit. The specimen is then loaded until failure, and $G_{IIC}$ is determined using

(5) $$\begin{array}{c} G_{IIC}=\frac{3mP_{max}^{2}a_{0}^{2}}{2B}, \end{array}$$

where $a_{0}$ is the delamination length and *B* is the width of the specimen.

### 3.5. Moisture Study

Moisture absorption equations developed by Shen and Springer were used to interpret the moisture uptake data collected from the traveller specimens [35]. Equation (6) was used to calculate the diffusivity, *D*, of IM7/8552,

(6) $$D=\pi {\left(\frac{h}{4M_{m}}\right)}^{2}{\left(\frac{M_{2}-M_{1}}{\sqrt{t_{2}}-\sqrt{t_{1}}}\right)}^{2},$$

where $M_{m}$ is the percent maximum moisture content, *M* is percent moisture content, *t* is time in days and *h* is half the specimen thickness.

A Fickian moisture model using a time-dependent parameter, $M_{F}$, was fitted using

(7) $$M_{F}\left(t\right)=M_{m}\left(1-exp\left[-7.3{\left(\frac{Dt}{h^{2}}\right)}^{0.75}\right]\right),$$

to demonstrate the Fickian behaviour of the material from the collected experimental data [28].

Dynamic Mechanical Analysis (DMA) was carried out using a DMA Q800 (TA Instruments, New Castle, Delaware, USA ) to determine the glass transition temperature, $T_{g}$, of the tested specimens. ‘Dry’ and ‘Wet’ specimens were tested following BS EN 6032:2015 [36]. $T_{g}$-onset was calculated using the storage modulus curve, and $T_{g}$-peak was determined from the maximum of the tan delta and loss modulus curves.

### 3.6. Post-Fracture Analysis

Fractography was employed using a scanning electron microscope (SEM) (Hitachi TM3030Plus tabletop Microscope, Tokyo, Japan) to examine the surfaces of the specimens after failure had taken place. This allowed the specimens to be analysed qualitatively and identify a brittle versus a ductile fracture surface, debonding between particles in the matrix, fibre bridging, plucking, and nesting [37]. Characteristics in surface morphology that signify varying fracture modes, failure initiation, crack propagation, test conditions and specimen moisture content are identified. The outlined features are key to understanding how a structure has failed [38].

## 4. Results and Discussion

### 4.1. Moisture Uptake and Thermal Analysis

**Figure 3:**
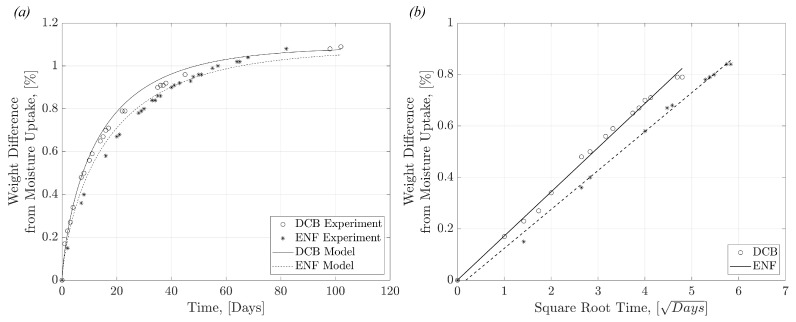
Plot of (**a**) moisture uptake over time, where ‘DCB Experiment’ and ‘ENF Experiment’ points are plotted using the recorded weight differences of the traveller specimens, and ‘DCB Model’ and ‘ENF Model’ are plotted using Equation (7), and (**b**) shows the linear fitted region for Fickian Response of moisture uptake against the square root of time, with points plotted using representative ‘DCB’ and ‘ENF’ specimens.

The moisture-conditioned ‘Wet’ specimens reached equilibrium saturation after a conditioning period of 3 months. The moisture uptake displayed by the specimens over time was recorded and is shown in Figure 3a. Full saturation was achieved at ≈1.1% weight gain from water, matching similar saturation levels seen in the literature [39]. The diffusivity, *D*, and maximum moisture content, $M_{m}$, for the studied specimens are shown in Table 2. Using these calculated values, a Fickian moisture model curve has been fitted to the experimental data collected, shown in Figure 3a, using Equations (6) and (7). Figure 3b shows the linear fitted region of the Fickian response, which was observed during the initial stages of moisture conditioning, up to ≈75% of $M_{m}$. The slopes of the linear region used to calculate *D* using Equation (6) were $1.72\times 10^{-3}$ ($R^{2}=0.991$) for the DCB specimen and $1.52\times 10^{-3}$ ($R^{2}=0.996$) for the ENF specimen. Using relatively high immersion temperatures for conditioning accelerates the diffusion of water molecules and the subsequent rate of moisture uptake within the material [40].

**Figure 4:**
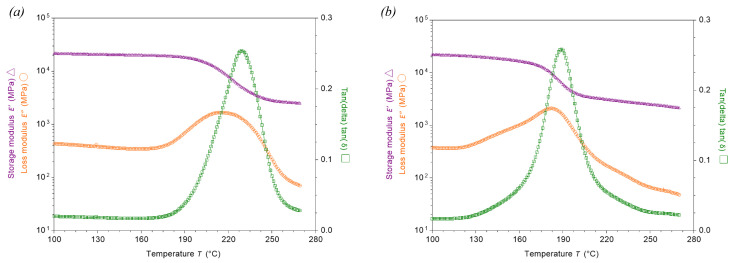
Storage Modulus, Loss Modulus and Tan Delta versus temperature in a (**a**) Dry specimen, and a (**b**) Wet specimen, obtained via DMA.

Figure 4 shows Storage Modulus, Loss Modulus and Tan Delta versus time for the ‘Dry’ and ‘Wet’ specimens. Table 3 shows the calculated $T_{g}$ for the tested specimens, extracted from the plotted curves. There is a 13% difference in $T_{g}$-onset (Storage Modulus), a 15% difference in $T_{g}$-peak (Loss Modulus) and a 17% difference in $T_{g}$-peak (Tan Delta) between ‘Dry’ and ‘Wet’ specimens. It is evident from the decrease in $T_{g}$ that plasticization and hydrolysis, the breaking of bonds due to water and subsequent decrease in cross-link density within the polymer, has taken place [41,42,43].

### 4.2. Mode I Interlaminar Fracture

**Figure 5:**
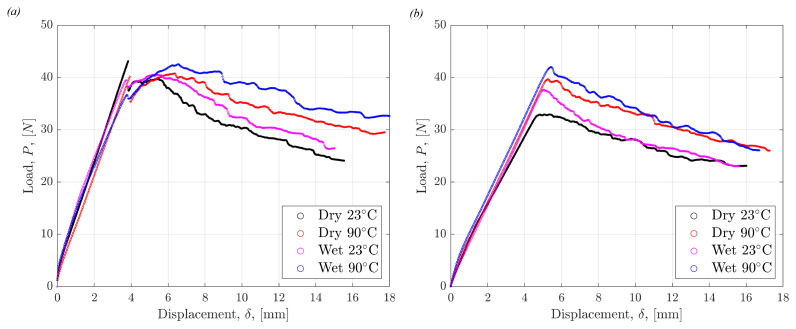
Representative load displacement curves in all tested conditions for DCB (**a**) non-precracked and (**b**) precracked specimens.

**Figure 6:**
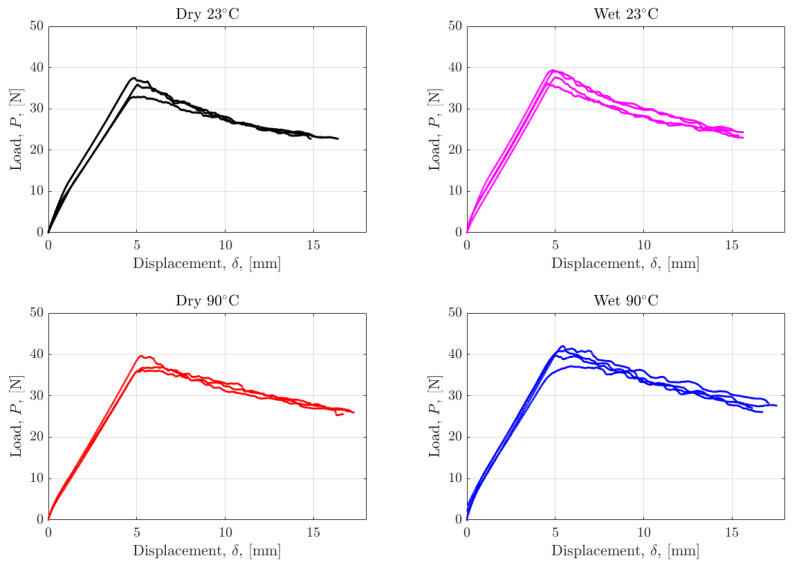
Load displacement curves in all tested conditions for all DCB precracked specimens.

Mode I fracture tests were carried out for all conditions using both non-precracked and precracked samples. Czabaj et al. have emphasized the need for precracking to create sharp starter cracks, which is supported by the findings in this work [44]. A larger force is required to initiate crack growth, and the behaviour immediately after initial crack growth is unstable in the non-precracked specimens, as shown in Figure 5a. This is predominantly due to the resin build-up around the crack tip created by the PTFE film insert. This creates a blunt, resin-rich region instead of a sharp crack tip, which inherently requires more force to propagate the crack. The evidence of this feature observed via fractography will be explored in Section 4.4. The variation between the load-displacement curves of the non-precracked specimens is larger compared to those that were precracked. The differences in Figure 5a,b highlight the importance of precracking to establish an infinitesimally small crack tip for fracture propagation. Figure 6 shows the load displacement curves for all tested precracked specimens. The load required to initiate and propagate the cracks through specimens was on average highest in the Wet 90 °C specimens and lowest in the Dry 23 °C specimens.

The following colour palette was applied consistently throughout the figures to represent each test condition to ease interpretation: ‘Dry 23 °C’ in black, ‘Wet 23 °C’ in magenta, ‘Dry 90 °C’ in red, ‘Wet 90 °C’ in blue.

**Figure 7:**
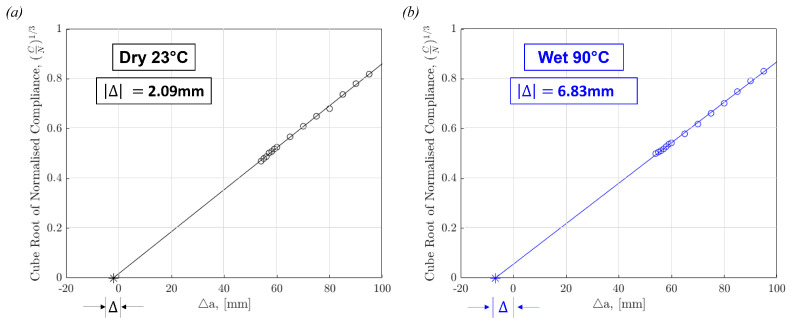
Compliance Calibration curves for representative DCB curves from (**a**) Dry 23 °C and (**b**) Wet 90 °C tests, with the x-axis intercept point representing |Δ| used in Equation (3).

Figure 7 shows two representative calibration curves for Dry 23 °C and Wet 90 °C used to obtain the correction factor, Δ, in Equation (3) which accounts for the compliance of the specimens. This was done for each specimen before calculating its respective interlaminar fracture toughness. The plots point to higher compliance in tests carried out at elevated temperatures and on wet-conditioned specimens. This finding supports the selection to use the Modified Beam Theory method for the calculation of $G_{IC}$, especially when testing at varying temperatures and moisture contents.

**Figure 8:**
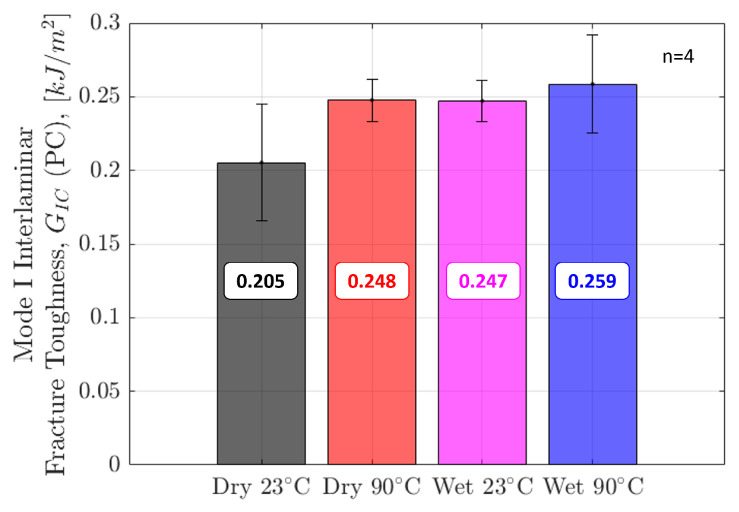
Mode I Initiation Interlaminar Fracture Toughness ($G_{IC}$) values obtained from precracked DCB specimens in all four environmental conditions.

Figure 8 shows the Mode I Interlaminar Fracture Toughness at the initiation of crack growth, $G_{IC}$, calculated using Equation (3). The critical force and displacement values used for the calculation were taken from the load-displacement curves shown in Figure 6. $G_{IC}$ is only reported for the precracked specimens with sharp crack tips. The $G_{IC}$ values of non-precracked specimens are roughly 30% higher than precracked specimens due to the dominating contribution from the blunt crack tip found in those specimens, as previously discussed.

As seen in Figure 8, $G_{IC}$ is lowest in the Dry 23 °C specimens and highest in the Wet 90 °C specimens. There was little difference in $G_{IC}$ between the Dry 90 °C and Wet 23 °C specimens, suggesting both moisture and elevated temperature have an equal contribution to the increase in the force required to initiate crack growth in unidirectional IM7/8552. There is a 26% increase in Mode I interlaminar fracture toughness in ‘hot/wet’ conditions compared to dry, room temperature conditions. These results show that IM7/8552 has higher fracture resistance in ‘hot/wet’ conditions when loaded in Mode I and point to the suitability of its use in parts where the prescribed elevated moisture and temperature conditions are experienced.

**Figure 9:**
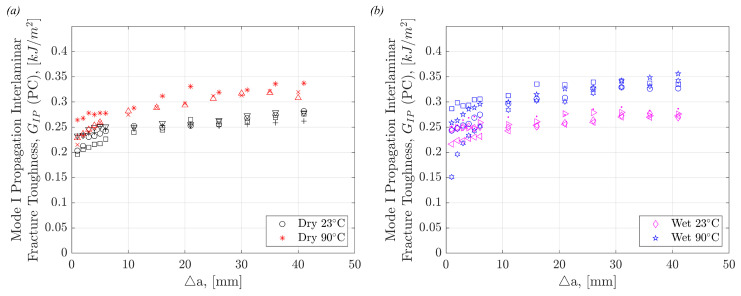
R-curves comparing (**a**) Dry 23 °C specimens versus Dry 90 °C specimens, and (**b**) Wet 23 °C specimens versus Wet 90 °C specimens.

Figure 9 shows the resistance to the propagation of a delamination crack, known as R-curves, in the various test conditions and moisture state. Figure 9a compares Dry specimens at room and elevated temperatures to show the significant difference between delamination resistance at the two temperatures. The difference is less significant at smaller crack lengths but is amplified as crack length increases. This pattern is also shown in Figure 9b where the R-curves of Wet specimens at room and elevated temperatures are compared. These results support what is known about the effect of moisture and temperature on OMCs. Matrix softening occurs due to the presence of moisture and heat, leading to a more ductile behaviour in the matrix. Fibre bridging and pull-out are therefore amplified in ‘hot/wet’ conditions. The visible effects leading to the differences in interlaminar fracture properties will be outlined in Section 4.4.

**Figure 10:**
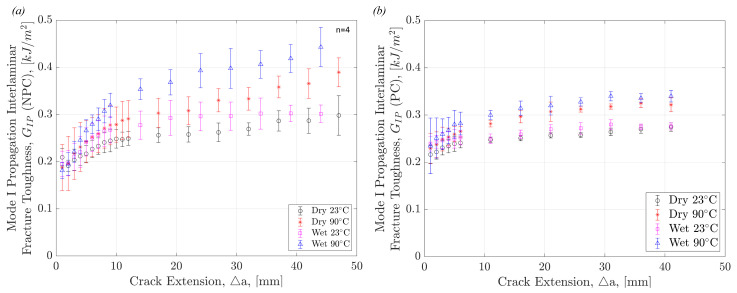
R-curves of all (**a**) non-precracked and (**b**) precracked DCB specimens, across all four environmental conditions.

The R-curves for all precracked and non-precracked Mode I tested specimens are shown in Figure 10. This shows that the effect of precracking is not only demonstrated in initiation values but also in consequent crack propagation. These plots help to highlight the extreme importance of not only precracking specimens, but also highlighting in data sets whether or not specimens have been precracked and by what method they were precracked. Figure 10 shows the same trend as Figure 8, with the R-curves demonstrating the highest resistance to delamination propagation in Wet 90 °C specimens and lowest in the Dry 23 °C specimens. However, unlike Figure 8, Figure 10 shows a significant difference between Dry 90 °C and Wet 23 °C specimens. There is a higher resistance to delamination in Dry 90 °C compared to Wet 23 °C. Figure 10 points out that temperature has a more significant effect than moisture on Mode I interlaminar crack propagation.

**Figure 11:**
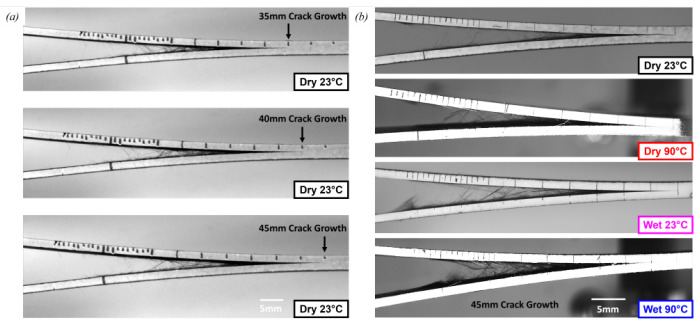
DCB specimens during testing in (**a**) Dry 23 °C conditions comparing different delamination lengths (35 mm, 40 mm, and 45 mm) and (**b**) all four environmental conditions at the same delamination length (45 mm).

Figure 11a shows a DCB specimen tested in Dry 23 °C conditions at 35 mm, 40 mm, and 45 mm crack growth. Fibre pull-out and fibre bridging are demonstrated and amplified at larger delamination lengths. This is a visual demonstration of how cracks are arrested by fibre-dominated mechanisms. Figure 11b compares DCB specimens from all test conditions after 45 mm of crack growth. The highest concentration of fibre bridging can be seen in the Wet 90 °C specimen. This gives insight into the cause of the high Mode I propagation interlaminar fracture toughness values.

### 4.3. Mode II Interlaminar Fracture

**Figure 12:**
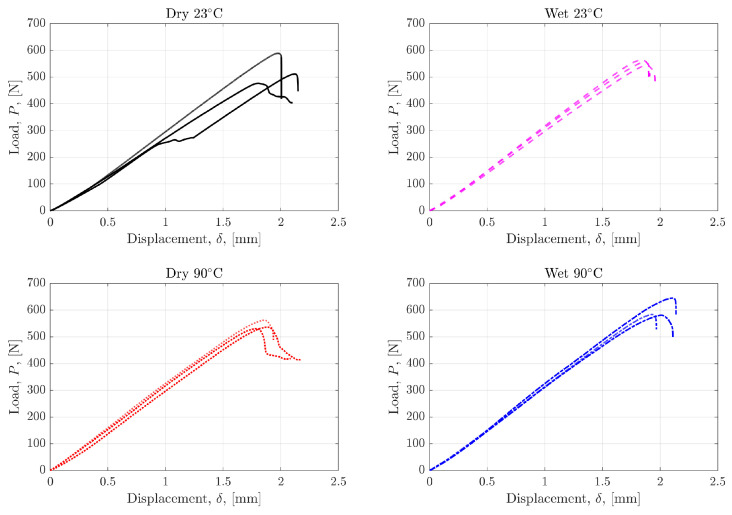
Load-displacement curves for precracked ENF specimens.

The load-displacement curves for the Mode II tests are shown in Figure 12. The drop in the load signified crack propagation and the conclusion of the test. One specimen from the Dry 23° batch demonstrates a momentary change in stiffness, as denoted by the constant load as displacement continued to increase around the 1 mm mark. This may be due to defects introduced during specimen preparation or slip whilst loading, resembling premature failure. Overall, the specimen repeatability was reliable.

**Figure 13:**
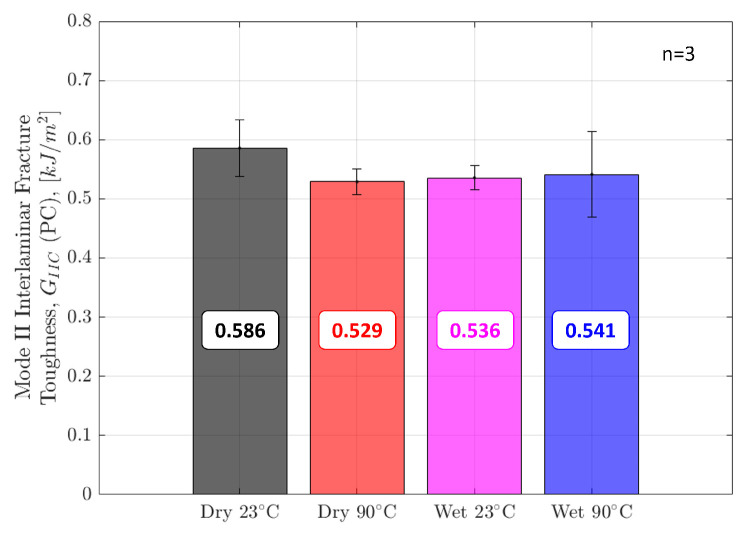
Mode II Initiation Interlaminar Fracture Toughness ($G_{IIC}$) for precracked ENF specimens in all four environmental conditions.

Figure 13 shows the Mode II interlaminar fracture toughness value for crack initiation in precracked specimens. Contrasting the Mode I results, Dry 23° specimens demonstrated the highest Mode II interlaminar fracture toughness. There is more scatter across Dry 23° and Wet 90° specimens. The average knock-down in fracture toughness from Dry 23° to Wet 90° is 8%. Elevated moisture and temperature have much less significance on Mode II compared to Mode I interlaminar fracture toughness. Only results for precracked specimens are shown. Work conducted on non-precracked specimens resulted in roughly 30% higher values compared to precracked specimens. Propagation values were not calculated in Mode II due to unstable crack growth.

### 4.4. Fractographic Analysis

**Figure 14:**
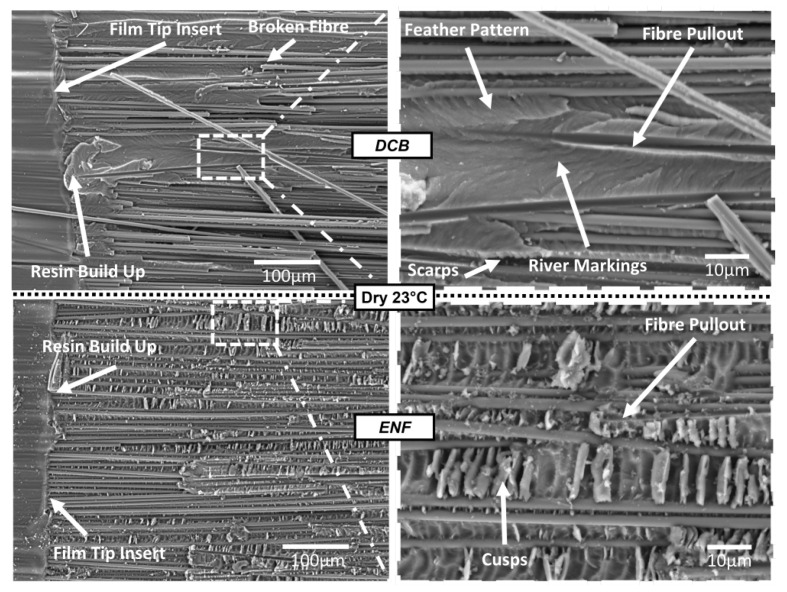
SEM image of the bottom surface of fractured specimens from Dry 23 °C DCB and ENF specimens. Image location: crack tip created by the PTFE film insert.

SEM images of the bottom surface of a DCB specimen and an ENF specimen from Dry 23 °C tests are shown in Figure 14. The location of the tip of the film insert can be seen, as well as the resin build-up in this region, discussed in Section 4.2. For precracked specimens, the specimens were initially cracked so the delaminations always passed through this region for more accurate results. Broken fibres can be seen on the surface of the specimen. Locations where fibres had pulled out of the surface are indicated. The DCB specimen contains matrix patterns including feather patterns, river marking and scarps, all indicating Mode I interlaminar fracture. The ENF specimen’s main identifiable feature is cusps, indicating Mode II interlaminar fracture.

**Figure 15:**
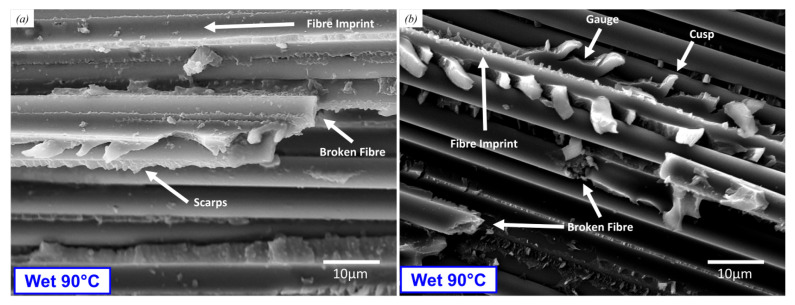
SEM image of the bottom surface of (**a**) a DCB specimen and (**b**) an ENF specimen, both tested in Wet 90 °C test. Image location: 20 mm from the PTFE film insert.

Figure 15 shows a highly magnified region from a DCB and an ENF specimen tested in the Wet 90° test condition. The location of these SEM images is 20 mm from the film insert tip. At this higher magnification, we can see the imprints of fibres that have been pulled out and fragments of the matrix that have debonded. Matrix patterns are clearly visible and demonstrate the direction of crack growth. Figure 15b shows clear evidence of broken fibres and fibre-matrix separation.

**Figure 16:**
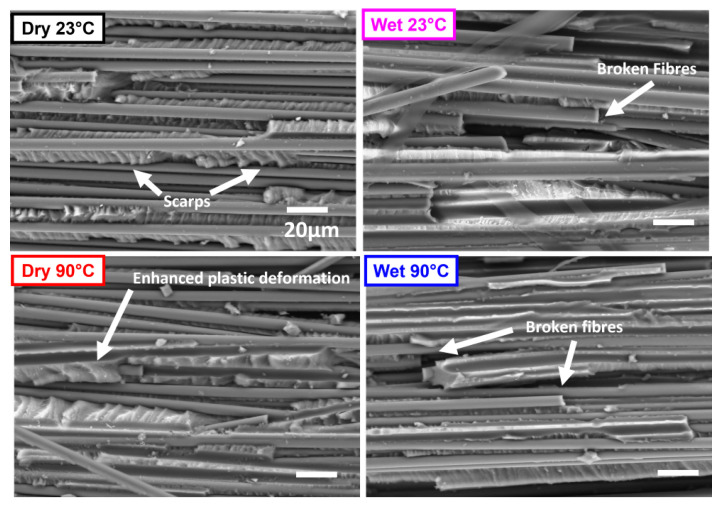
SEM image comparing the bottom surface of a fractured specimen from Mode I interlaminar fracture test in Dry 23 °C, Wet 23 °C, Dry 90 °C and Wet 90 °C specimens. Image location: 10 mm from the PTFE film insert.

Figure 16 compares DCB specimens from each of the test conditions, with images taken 10 mm from the film insert. The large concentration of scarps, the river lines, and the textured matrix flow seen in the ‘Dry 23 °C’ specimens demonstrate brittle failure. There are fewer scarps on the ‘Wet’ compared to ‘Dry’ specimens, demonstrating a more ductile matrix, which resulted in slower delamination growth. Enhanced plastic deformation is noted in the ‘Dry 90 °C’ specimens due to the softening of the matrix, which also resulted in increased ductility and slower delamination growth as seen in $G_{IC}$ propagation values. There is a local rotation of a broken fibre and its attached matrix in the ‘Wet 90 °C’, demonstrating the matrix carrying the fibre during delamination. The enhanced fibre bridging in this test condition resulted in fibre pull-out and noted resulting fibre imprints on the fractured surface. There is an overall decrease in blunt matrix features between ‘Dry’ and ‘Wet’ specimens, pointing to more plastic behaviour.

**Figure 17:**
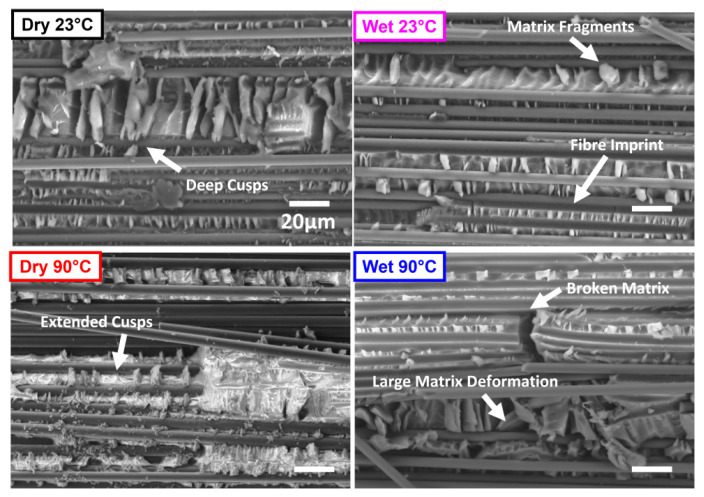
SEM image comparing the bottom surface of fractured specimens from Mode II interlaminar fracture test in Dry 23 °C, Wet 23 °C, Dry 90 °C and Wet 90 °C specimens. Image location: 20 mm from the PTFE film insert.

Figure 17 compares ENF specimens from each of the test conditions, with images taken 20 mm from the film insert. Cusps are clearly defined in all cases, and are slightly more dominant in the Dry specimens compared to the Wet specimens. In the ‘Dry 23 °C’ specimen, the cusps are tall, large, and sharp, pointing to the brittle nature of this fracture. In the ‘Wet’ specimens, there are noted fibre imprints and evidence of ‘clean’ fibres with little matrix on the fibres, pointing to a degraded fibre-matrix interface due to moisture. Matrix fragments are also observed, detached from the specimen during fracture. The cusps are more drawn out, demonstrating a more plasticised matrix. There are extended cusps in the ‘Dry and Wet 90 °C’ specimens as the matrix softened with increased temperature. There are large, multidirectional matrix deformations in the ‘Wet 90 °C’ specimen, which demonstrates the synergistic effect of heat and moisture on the plasticisation and softening of the matrix. Moisture-induced fibre-matrix deterioration and heat-induced softening cause reductions in $G_{IIC}$ when applied individually. However, the combination of the two, enhanced by the swollen plasticised matrix, produced more scattered $G_{IIC}$ results.

The combined effect of increased heat and humidity on IM7/8552 ultimately led to an increase in $G_{IC}$ due to the plasticisation of the matrix, resulting in increased ductility, matrix softening and fibre bridging. The effect was the opposite on $G_{IIC}$ due to the detrimental effects of moisture and temperature on the fibre-matrix interface.

## 5. Conclusions

This work has characterised the Mode I and Mode II interlaminar fracture toughness of IM7/8552, a high-temperature carbon/epoxy composite, in four different environmental conditions. Specimens were conditioned to be Dry or Wet (ASTM D5229 [34]) and tested at 23 °C and 90 °C, resulting in the four test conditions: Dry 23 °C, Wet 23 °C, Dry 90 °C, and Wet 90 °C.

- IM7/8552 has been shown to exhibit a Fickian Response to moisture uptake. A maximum moisture content of ≈1.1% was achieved after ≈100 days of conditioning in 70 °C/85% relative humidity.
- The glass transition temperature of the material decreased by 13% ($T_{g}$-onset, Storage Modulus), 15% ($T_{g}$-peak, Loss Modulus) and 17% ($T_{g}$-peak, Tan Delta) in ‘Wet’ specimens compared to ‘Dry’, characterised by DMA.
- Mode I initiation and propagation interlaminar fracture toughness values were calculated using DCB specimens, and R-curves were plotted to show the increase in fracture toughness as delamination increased. Mode I toughness was highest in the ‘hot/wet’ (Wet 90 °C) specimens, where toughness values were on average 26% higher than Dry 23 °C specimens.
- Temperature was more dominant than moisture in increasing Mode I fracture toughness as delamination progressed, as evidenced by the R-curves. This is due to matrix softening, resulting in more compliant specimens and an increase in the ductile behaviour of the composite. Fibre bridging and pull-out are also enhanced, further arresting crack growth.
- Mode II initiation fracture toughness values were calculated using ENF specimens. Mode II toughness was highest in Dry 23 °C specimens, reducing by 8% in ‘Wet 90 °C’. ENF specimens tested in ‘hot/wet’ conditions had the largest scatter.
- Temperature and moisture contributed to an increase in Mode I interlaminar fracture toughness and a decrease in Mode II interlaminar fracture toughness.
- The strong influence of the resin build-up in the film tip region is displayed using SEM images, highlighting the importance of precracking before testing.
- Mode I fracture contains matrix features including feather patterns, river markings and scarps. Mode II fracture is identified by matrix cusps. Both types of fracture demonstrate evidence of broken fibres and fibre pull-out.
- Blunt fracture behaviour was noted in Dry 23 °C specimens, demonstrated by sharp, highly concentrated scarps (Mode I) and deep, sharp cusps (Mode II).
- Extended matrix deformation with smoother, drawn-out features was observed in high-temperature tests. Clean fibres with less resin residue pointed to a degraded fibre-matrix interface caused by moisture in ‘Wet’ specimens
- ‘hot/wet’ conditions resulted in a ductile, soft, plasticised matrix with broken fibres, large local deformations and deposited matrix fragments.

Using the techniques and processes detailed in this study, work is ongoing to characterise novel OMCs containing cyanate ester matrices. Higher resolution SEM analysis is being carried out to analyse surface features and gain a better insight into the effect of moisture and temperature on surface features.

## Figures and Tables

**Table 1:** Mechanical Properties of a Typical Composite composed of 0° IM7 (12k) fibres and HexPly^®^ 8552 Epoxy Resin [29].

Tensile Strength	Tensile Modulus	Tensile Strain	Short Beam Shear Strength	Compressive Strength	Compressive Modulus
2723 MPa	164 GPa	1.62%	137 MPa	1689 MPa	150 GPa

**Table 2:** Diffusivity, *D*, [${\mathrm{mm}}^{2}/\mathrm{Day}$] and Maximum Moisture Content, $M_{m}$, [%] calculated from the moisture uptake study of DCB and ENF specimens.

Specimen	*D*, [mm^2^/Day]	$M_{m}$, [%]
DCB	$1.584\times 10^{-2}$	1.09
ENF	$1.257\times 10^{-2}$	1.08

**Table 3:** $T_{g}$ calculated using Storage Modulus, Loss Modulus and Tan Delta for ‘Dry’ and ‘Wet’ specimens, obtained via DMA.

Condition	$T_{g}$-Onset [°C] (Storage Modulus)	$T_{g}$-Peak [°C] (Loss Modulus)	$T_{g}$-Peak [°C] (Tan Delta)
Dry	202	213	229
Wet	175	182	189

## Data Availability

The original contributions presented in this study are included in the article. Further inquiries can be directed to the corresponding author.
